# Characteristics of Pyroptosis-Related Subtypes and Novel Scoring Tool for the Prognosis and Chemotherapy Response in Acute Myeloid Leukemia

**DOI:** 10.3389/fonc.2022.898236

**Published:** 2022-06-10

**Authors:** Jingjing Pan, Yinyan Jiang, Changhong Li, Ting Jin, Kang Yu, Zhenlin Jin

**Affiliations:** ^1^ Department of Laboratory Medicine, The First Affiliated Hospital of Wenzhou Medical University, Wenzhou, China; ^2^ Department of Hematology, The First Affiliated Hospital of Wenzhou Medical University, Wenzhou, China; ^3^ Department of Operating Room, The First Affiliated Hospital of Wenzhou Medical University, Wenzhou, China

**Keywords:** acute myeloid leukemia, pyroptosis, molecular subtype, tumor microenvironment, prognosis, therapeutic sensitivity

## Abstract

Acute myeloid leukemia (AML) is usually associated with poor prognosis and low complete remission (CR) rate due to individual biological heterogeneity. Pyroptosis is a special form of inflammatory programmed cell death related to the progression, treatment response, and prognosis of multiple tumors. However, the potential connection of pyroptosis-related genes (PRGs) and AML still remains unclear. We described the genetic and transcriptional alterations of PRGs in 151 AML samples and presented a consensus clustering of these patients into two subtypes with distinct immunological and prognostic characteristics. Cluster A, associated with better prognosis, was characterized by relatively lower PRG expression, activated immune cells, higher immune scores in the tumor microenvironment (TME), and downregulation of immunotherapy checkpoints. Subsequently, a PRG score was constructed to predict overall survival (OS) of AML patients by using univariate and multivariate Cox regression analysis, and its immunological characteristics and predictive capability were further validated by 1,054 AML samples in external datasets. Besides an immune-activated status, low-PRG score cohorts exhibited higher chemotherapeutic drug sensitivity and significant positive correlation with the cancer stem cell (CSC) index. Combined with age, clinical French-American-British (FAB) subtypes, and PRG score, we successfully constructed a nomogram to effectively predict the 1-/3-/5-year survival rate of AML patients, and the predictive capability was further validated in multiple external datasets with a high area under the curve (AUC) value. The various transcriptomic analysis helps us screen significant pyroptosis-related signatures of AML and provide a new clinical application of PRG scores in predicting the prognosis and benefits of treatment for AML patients.

## Introduction

Acute myeloid leukemia (AML) is the most common malignant hematologic cancer with high morbidity and mortality, leading to a poor prognosis and an increasing social burden (1). The current mainstream therapeutic regimen involves intensive induction chemotherapy (7 + 3 regimen), hypomethylated (HMA) drugs, and hematopoietic stem cell transplantation (2). The complete remission (CR) rate of AML usually arrives at only 50% after conventional treatment, but more than 20% of AML patients still remain resistant. Even among patients with CR, approximately 50% of cases exhibited disease recurrence due to its high degree of genetic heterogeneity including various genetic mutations and complex molecular subtypes (3). Despite remarkable progress has been improved for the treatment of AML, especially chimeric antigen receptor T (CAR-T) cell therapy, there are still insurmountable obstacles for its wide clinical practice such as drug resistance, immune escape, and graft-versus-host response (4). Therefore, it is crucial to screen reliable biomarkers and convenient molecular phenotypes to predict outcomes, especially the curative effects for AML patients.

Pyroptosis is a special form of inflammatory programmed cell death (PCD) characterized by cleaving the gasdermin D (GSDMD) through classical or non-classical pathways and triggering the release of cell contents [including inactive cytokines like interleukin (IL)-18 and IL-1β] to induce a strong inflammatory response (5). Different from apoptosis, pyroptosis usually occurs faster and inflammatory components are associated with the promotion of tumor relapse and angiogenesis. In addition, pyroptosis was also reported to construct a tumor-suppressive microenvironment *via* releasing inflammatory factors and chemotherapeutic drugs could play antitumor effects through inducing the pyroptosis of multiple tumor cells, such as colorectal cancer, breast cancer, and thyroid cancer (6–8). Johnson et al. (9) also demonstrated that dipeptidyl peptidase 8/9 (DPP8/DPP9) inhibitors could induce pyroptosis to ameliorate AML *via* pharmacological intervention experiments *in vitro*. However, the exact relationship of pyroptosis with the molecular phenotype, therapeutic response to chemotherapy, and prognosis of AML remains unclear.

The risk stratification of AML patients based on transcriptome RNA profiles *via* high-throughput sequencing has been considered as a novel technique that can quickly reveal biological characteristics and help us to identify the most appropriate treatment strategies (10). Besides conventional transcriptome sequencing, multifarious biological characteristics have also been applied to investigate novel molecular phenotypes for the prognosis of AML, such as immune microenvironment (11), autophagy-related signatures (12), and N6‐methyladenosine (13). In this study, we comprehensively estimated the genetic and transcriptive characteristics of pyroptosis-related genes (PRGs) in AML patients and stratified the cohorts into two discrete subtypes based on their expression. The intratumoral immune landscape and clinical prognostic signatures of pyroptosis-related subtypes were further expounded including the tumor microenvironment (TME), immune cell infiltration (ICI), and immune checkpoint analysis. Subsequently, a novel index called PRG score was further constructed based on pivotal PRGs, and a useful scoring system that combined PRG scores with other classical clinical features was successfully established to improve the prognostic risk stratification and facilitate making an accurate treatment decision for AML patients.

## Materials and Methods

### Acute Myeloid Leukemia Dataset Collection and Preprocessing

Transcriptome profiling data (fragments per kilobase million/FPKM value) of 151 AML bone marrow (BM) samples with their corresponding clinical data were downloaded from The Cancer Genome Atlas (TCGA) datasets (https://portal.gdc.cancer.gov/). The RNA sequencing (RNA-seq) dataset of 70 normal BM samples was downloaded through the GTEx database (14). In addition, other microarray datasets of AML patients with prognostic information were obtained from the Gene Expression Omnibus (GEO) datasets (https://www.ncbi.nlm.nih.gov/geo/), including 250 AML samples in GSE106291, 242 AML samples in GSE12417, and 562 AML samples in GSE37642. Notably, survival outcomes of TCGA-LAML patients from Cancer and Leukemia Group B (CLAGB 8461) were obtained from the UCSC Xena platform (https://xenabrowser.net/datapages/), and therapeutic response to primary chemotherapy and information on Runx1 were gained from the above GEO datasets. All of these datasets fulfilled the following inclusion criteria: 1) using the key words of “Acute Myeloid Leukemias” or “Leukemias, Acute Myeloid”; 2) mRNA expression data of RNA-seq or microarray from BM tissues; 3) the number of samples in each dataset is more than 10. The exclusion criteria included that the following: 1) patients with other severe systemic diseases or hematonosis; 2) samples lacking corresponding clinical characteristics for analysis, such as survival outcomes and pathological stages. For detailed information on these datasets, refer to **Supplementary Table S1**. The “sva” package with “ComBat” algorithm was further applied to remove the technical biases due to batch effects between TCGA and GTEx datasets (15).

### Mutational and Expressional Characteristics of Pyroptosis-Related Signatures in Acute Myeloid Leukemia Patients

According to previous studies, 33 pyroptosis-related signatures were chosen in our study including the Caspase families (CASP) (16, 17), Gasdermin families (GSDM) (18), Granzyme families (GZM) (19), inflammasome-associated families [nucleotide-binding domain and leucine-rich repeat receptor (NLR)], and special inflammatory factors (IL1β and IL18) (20, 21). After filtering the invalid gene with low expression (mean FPKM value <1), a total of 31 pyroptosis-related signatures were screened and their corresponding mutation annotation format (MAF) was further obtained from the UCSC Xena platform including somatic mutation and copy number variants (CNVs). The “maftools” package (22) was applied to exhibit the somatic mutation of PRGs, and “RCircos” package (23) was used to display their CNV and location on different chromosomes.

### Consensus Cluster Analysis for Pyroptosis-Related Signatures in Acute Myeloid Leukemia

Based on the expression of PRGs, the “ConsensuClusterPlus” package (24) was applied to perform the unsupervised clustering based on Spearman distance and hierarchical methods with 1,000 repeated times (80% of samples each time) to ascertain the classification stability. In this process, we divided the AML patients into different clusters from 2 to 9 and the optimal clustering number was determined with the optimal consensus cumulative distribution function (CDF) plot. In addition, we also performed multiple comparisons among different pyroptosis subtypes including clinical FAB subtypes, CLAGB phenotypes, and expression of PRGs to explore their characteristics. Finally, the R package “survminer” (25) and “survival” (26) were used to conduct the Kaplan–Meier survival analysis and draw survival curves among pyroptosis subtypes.

### Immune Characteristics of Different Clusters in Acute Myeloid Leukemia

To evaluate the immunological features of pyroptosis-related clustering, we further performed the integrated analysis based on multiple immune aspects, including gene set variation analysis (GSVA), TME, ICI, and immune checkpoint analysis. Using the “c2.cp.kegg.v6.2.symbols” datasets downloaded from the MSigDB database, we performed GSVA based on the “GSVA” package and the heatmap was applied to exhibit the difference of pathways (27). For the TME analysis, the ESTIMATE algorithm was applied to evaluate the stromal and immune scores and tumor purity of each patient (28). Moreover, the “CIBERSORT” package (29) was used to quantitatively analyze the infiltration levels of 22 different human immune cells by 1,000 random permutations. To estimate the potential curative response to immunotherapy, we also compared the expression of immune checkpoints between pyroptosis-related subtypes including PD1/CD274, PD-L1/PDCD1, CTLA4, HAVCR2, and LAG3.

### Identification of Differentially Expressed Genes and Functional Enrichment Analysis Between Clusters

To better standardize datasets, the “DESeq” function of DESeq2 package was applied to perform the difference analysis. The differentially expressed genes (DEGs) between pyroptosis subtypes were identified using the “DESeq2” package with the significance cutoff as p value <0.05 and absolute fold change >1 (30). To further explore the biological function and characteristics of pyroptosis clusters, the Kyoto Encyclopedia of Genes and Genomes (KEGG) enrichment analysis was performed and visualized graphically by the “ClueGO” plugin in Cytoscape software (31).

### Establishment of the Pyroptosis-Related Gene Score

Subsequently, we screened seven common PRGs from DEGs between clusters in AML patients. To further define a novel parameter reflecting the prognostic characteristics of pyroptosis subtypes, we performed univariate Cox proportional hazards regression analysis for overall survival (OS) *via* the “coxph” function in “survival” package. Signatures with prominent prognostic worth were further put into multivariate Cox regression (stepwise model) to obtain the regressive coefficients, and the PRG score was established based on the following formula:


PRGscore=Exp(Gene1)∗β1+Exp(Gene2)∗β2+⋯+Exp(Genen)∗βn

where *Exp*(Gene) means the expression FPKM value of Gene, and β means the corresponding regression coefficient. The PRG score of every AML patient was calculated separately, and the cohorts were further divided into high- and low-PRG score subgroups using the median value as the cutoff value. Conformably, we also made similar comparisons between high- and low-PRG score groups including survival analysis, clinical phenotypes, TME, ICI, immune checkpoint, and pyroptosis-related signatures.

### Relationship of Microsatellite Instability, Cancer Stem Cell Index, Drug Susceptibility, and Pyroptosis-Related Gene Scores

As a novel prognosis index in oncological studies, microsatellite instability (MSI) scores were obtained from TCGA datasets and the mRNA stem index (mRNAsi) was obtained from Tathiane’s article (32), which reflect their correlation with multiple tumors’ prognosis and curative effects. Then, we compared the difference of MSI scores between high- and low-PRG score subgroups and performed the correlation analysis of mRNAsi and PRG scores with Spearman’s algorithm. For the analysis of drug susceptibility, AML patients with therapeutic reaction to initial chemotherapy (from GSE106291) were used to investigate the relationship between therapeutic response and PRG scores. Moreover, to further evaluate the concrete therapeutic value of PRG scores in the chemotherapy for AML, we calculated the half-maximal inhibitory concentration (IC50 value) of common chemotherapeutic drugs using the Genomics of Drug Sensitivity in Cancer (GDSC) databases (33). Chemotherapeutic drugs targeted to AML such as cytarabine, mitoxantrone, and methotrexate have been widely recommended for AML treatment by current clinical guidelines. Therefore, a comparison of these chemotherapeutic drugs’ IC50 value between PRG score subgroups was performed using Wilcoxon test, with the results exhibited in box-line diagrams by the “ggpubr” package (34).

### Construction and Validation of a Pyroptosis-Related Nomogram Scoring System

The multivariate Cox regression analysis (stepwise model) was applied to construct the prognostic nomogram scoring system for AML patients combined with PRG scores and other clinical characteristics, including age and FAB subtypes. Selected variables were identified as p values <0.05 or determined based on clinical practice, and the nomogram scoring system was further constructed to predict the probability of 1-, 3- and 5-year survival in AML patients using the “rms” package. To estimate and validate the prediction efficiency of the nomogram scoring system, we further plotted the calibration curves in its 1-, 3-, and 5-year survival through a bootstrapping method with 1,000 resamples. Moreover, time-dependent receiver operating characteristic (ROC) curves from other external GEO datasets were applied to assess the nomogram for 1-, 3-, and 5-year survival.

### Clinical Sample Collection and RT-qPCR Validation

A total of 15 BM samples of incipient AML patients were obtained from the First Affiliated Hospital of Wenzhou Medical University to perform quantitative reverse transcription polymerase chain reaction (RT-qPCR) experiments. All samples were conserved in RNAlater^®^ within -80°C, and then total RNA was isolated from tissues using TRIzol^®^ reagent (Invitrogen). Mean 1 μg of total RNA was used for the reverse transcription using the GoTaq^®^ Two-Step RT-qPCR System (Promega). For each PCR process, after enzyme activation at 95°C for 2 min, 40 cycles of amplification at 95°C was performed and completed after 60°C for 60 s. For each example, the PCR was repeated three times, and the gene expression of vital PRGs was measured according to the comparative ^△^Ct method. Subsequently, PRG scores of each sample were also well calculated, and the prognostic capacity was also validated for AML patients. The concrete clinical characteristics of these patients were also recorded, including age, gender, onset time, and genetic mutation, to evaluate the prognosis status by two experienced hematologists.

### Validation of Prognostic Capability Based on External Datasets

To validate the prognostic capability of PRG scores in external datasets, we also calculated corresponding PRG scores of five GEO datasets and performed Kaplan–Meier survival analysis. We also investigated the correlation of PRG scores and the Runx1’s mutation and fusion, which reflected the prognosis of AML patients. In addition, based on the results of RT-qPCR, we also calculated the PRG score of recruiting patients and validated the correlation of PRG scores and the clinical prognosis status. To further estimate the efficacy of PRG scores for AML, we also screened four other established scores to construct the predictive models and performed the time-dependent ROC analysis, including TME scores (35), Autophagy scores (36), Ferroptosis scores (37), and m6A-related long non-coding RNA (lncRNA) scores (38) (**Supplementary Table S19**).

### Ethics Statement and Statistical Analysis

All participants received a written informed consent for their enrollment, and this study was approved by the Ethics Committee of the First Affiliated Hospital of Wenzhou Medical University (Issuing Number: 2021063). All of the statistical analyses were performed in R software version 3.6.1 (https://www.r-project.org/). The Wilcoxon rank-sum test was used to compare continuous variables, and the Kaplan–Meier algorithm was applied to perform survival analysis. The two-tailed p value <0.05 was considered statistically significant.

## Results

### Genetic Degeneration and Functional Characteristics of Pyroptosis-Related Genes in Acute Myeloid Leukemia

**Figure 1:**
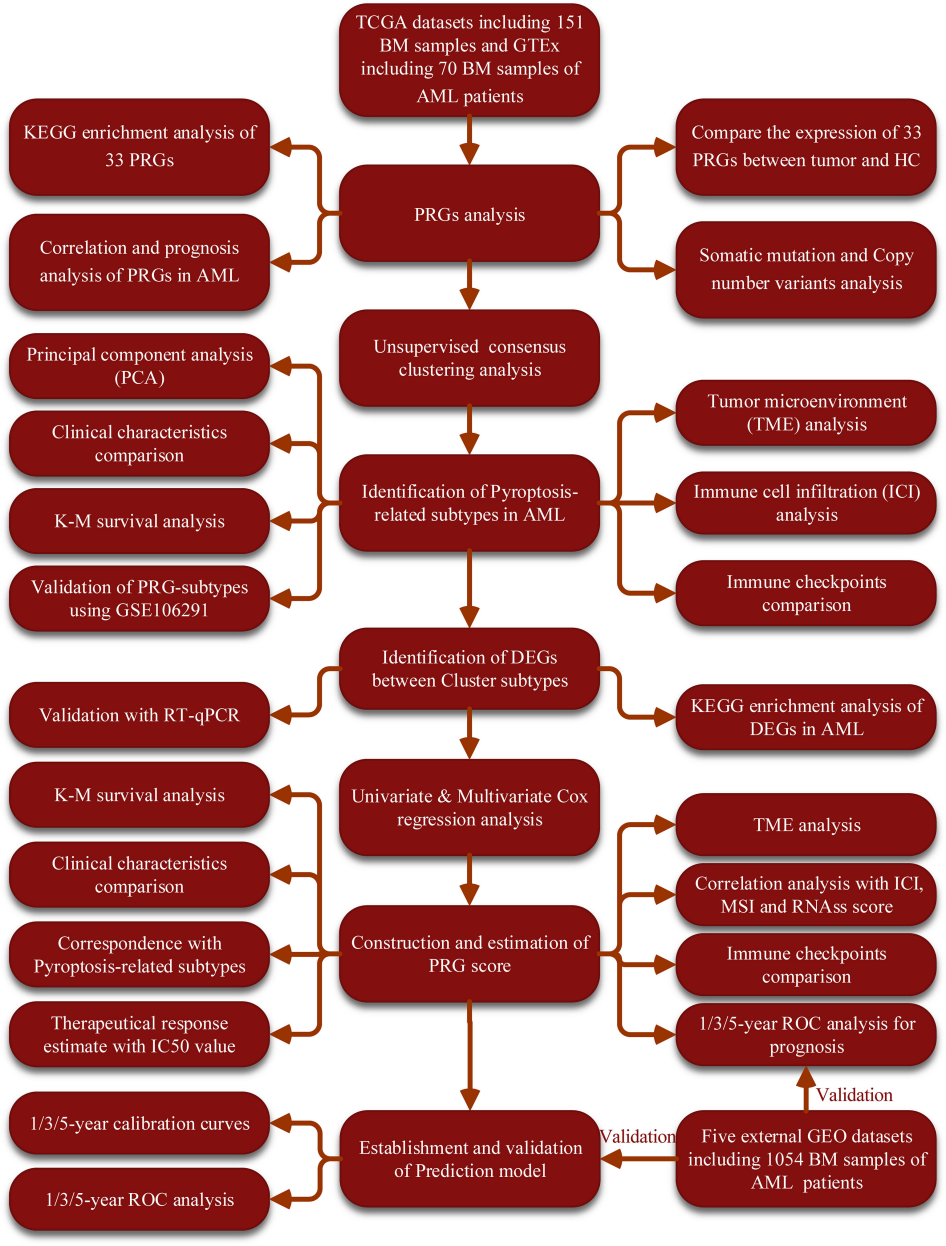
The flow diagram of the workflow in this study. BM, bone marrow; AML, acute myeloid leukemia; PRGs, pyroptosis-related genes; PCA, principal component analysis; CNV, Copy number variants; TME, tumor microenvironment; ROC, receiver operating characteristic; IC50, half-maximal inhibitory concentration; ICI, Immune cell infiltration; MSI, microsatellite instability.

**Figure 2:**
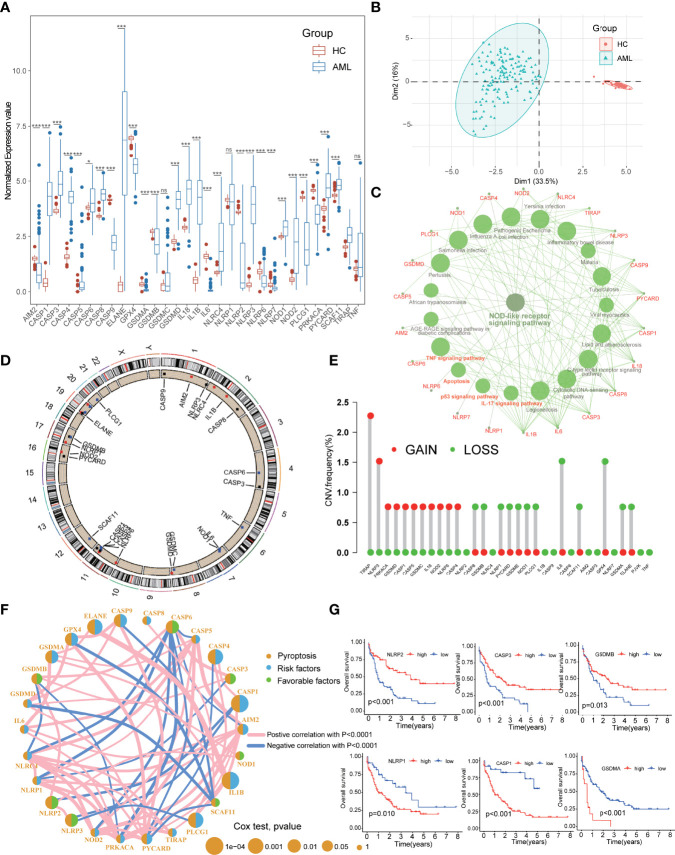
Expressional and mutational characteristics of PRGs in AML. **(A)** Expression of 33 PRGs between AML and the healthy control (HC). **(B)** PCA showing that the HC and the AML were significantly divided into two groups. **(C)** The KEGG pathway enrichment analysis of the PRGs. **(D, E)** The situation of CNV gain and loss of the PRGs on 23 chromosomes. **(F)** Prognostic characteristics and expressional relation among PRGs in AML**. (G)** PRGs with the same families but different outcomes for prognosis of the AML. AML, acute myeloid leukemia; PRGs, pyroptosis-related genes; PCA, principal component analysis; CNV, Copy number variants. *P<0.05, **P<0.01, ***P<0.001; ns, not significant.

The workflow of this study was summarized in **Figure 1**. The expression levels of the 33 PRGs were compared in TCGA and GTEx datasets from 70 normal and 151 tumor samples (**Supplementary Table S2**). It revealed that most PRGs were significantly upregulated in AML groups, such as CASP1/3/4/5/6/8, ELANE, GSDMD, IL18, IL1B, and NOD1/2 (**Figure 2A**). Principal component analysis (PCA) shows that the healthy control (HC) and AML patients were significantly divided into two groups based on these PRGs (**Figure 2B**), and the functional enrichment analysis indicated that these PRGs were enriched in immune-related pathways, such as NOD-like receptor signaling pathway, IL-17 signaling pathway, and TNF signaling pathway (**Figure 2C**). In addition, mutational analysis showed that somatic mutation of PRGs was detected in only 3% of cases, and the maximum CNV frequency was only 2.5% for PRGs (**Figures 2D, E**; **Supplementary Figure S1A**; **Supplementary Table S2**). The comprehensive situations of PRG’s mutual relation, regulator affiliations, and their prognostic values in AML patients were displayed in a network (**Figure 2F**; **Supplementary Tables S4, 5**). Interestingly, we discovered some PRGs from the same families, but they exhibited different outcomes for the prognosis of AML patients (**Figure 2G**).

### Pyroptosis-Related Subtypes and Their Clinical Characteristics in Acute Myeloid Leukemia

**Figure 3:**
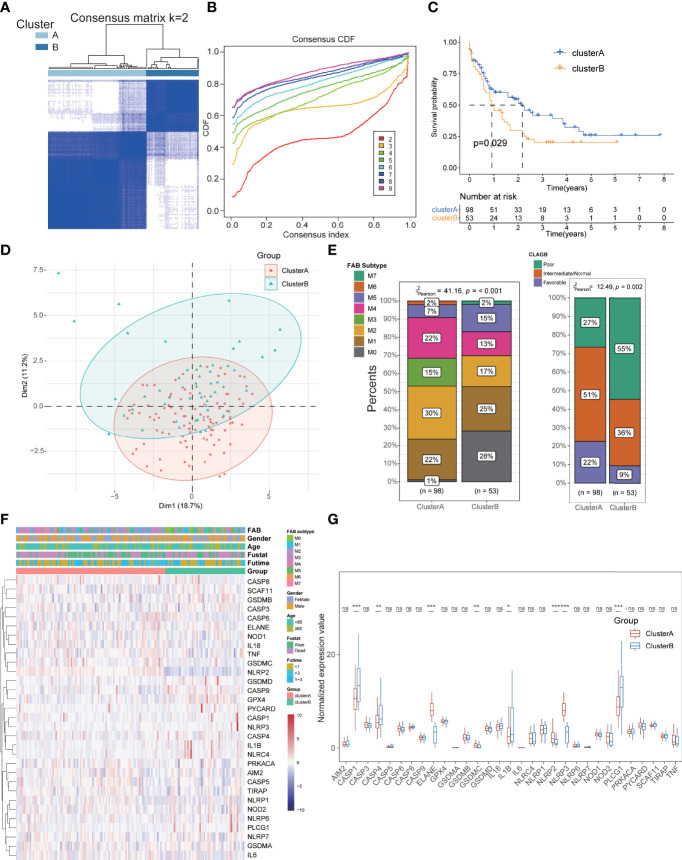
Identification of pyroptosis-related subtypes and clinical features in AML. **(A, B)** The cases could be well grouped into two clusters. **(C)** The survival analysis exhibited a better prognosis in cluster A than that in cluster B groups**. (D)** PCA showing significant differences in the pyroptosis-related transcription profiles between two clusters**. (E)** Cluster A patients manifested significant association with benign clinical characteristics than cluster B patients. **(F, G)** Comparison of the expression levels of these PRGs between two subgroups. *P<0.05, **P<0.01, ***P<0.001; ns, not significant.

To further explore and compare the regional characteristics of PRGs in AML, we constructed a consensus clustering analysis with all AML patients in TCGA cohort based on the expression of 33 PRGs. By investigating the clustering variable (k) alteration from 2 to 9 clusters, we found that k = 2 was the most optimal threshold with the maximal distribution of cumulative distribution function (CDF) and Delta area values, suggesting that the cases could be well grouped into two clusters (**Figures 3A, B**; **Supplementary Figure S1B**). Other improper cluster subtypes were shown in **Supplementary Figure S1B** with misty differences among groups, and the concrete clinical information of two clusters was displayed in **Table 1**. Survival analysis exhibited better prognosis in cluster A than that in cluster B groups (p = 0.029, **Figure 3C**), and significant differences in the pyroptosis-related transcription profiles between two clusters were demonstrated by PCA (**Figure 3D**). Interestingly, compared to the cluster B cohorts, cluster A patients manifested significant association with benign clinical characteristics, such as younger age, FAB subtypes sensitive to treatment (M3), and favorable outcomes of CLAGB (**Figure 3E**, **Supplementary Figure S1C**). Moreover, we also compared the expression levels of these PRGs between two subgroups, and the results revealed that several PRGs were downregulated in cluster A cohorts including CASP1, CASP4, IL1B, and PLCG1, while ELANE, NLRP2, and NLRP3 were upregulated in cluster A patients (**Figures 3F, G** **)**. To further validate the pyroptosis-related subtypes, the transcription profiles of 422 other AML patients in GSE37642 training datasets were applied to reperform the consensus clustering analysis, and it successfully demonstrated the existence of two subtypes with optimal CDF in AML patients (**Supplementary Figures S2A, B, F, G**). Consistent with the results of TCGA datasets, cluster A patients exhibited better survival outcomes, milder clinical stages, and lower PRG scores than those of cluster B cohorts (**Supplementary Figures S2C–E**). Based on the Least Absolute Shrinkage and Selection Operator (LASSO) and logistical regression analysis, we ultimately identified 4 key genes to construct the diagnostic model for cluster subtypes in AML, and the nomogram was used to predict the individual clusters, including KIR2DL1, LINC02805, OR52N5, and ERC2 (**Supplementary Figures S2H, I**). Interestingly, we also explored the diagnostic capacity of these key genes in cluster subtypes of AML patients, and it revealed that the combined genes exhibited a preeminent role with mean AUC values 0.845 in TCGA-LAML datasets and 0.764 in GSE37642 datasets (**Supplementary Figure S2J**).

**Table 1:** Comparison of clinical information between pyroptosis-related subtypes in AML patients.

Variables	Cluster A (n = 98)	Cluster B (n = 53)	p value (Chi-square test)
Age (n/%)			0.037*
≥65	24/24.49%	20/37.74%	
<65	74/75.51%	33/62.26%	
Gender (n/%)			0.964
Women	44/44.90%	24/45.28%	
Men	54/55.10%	29/54.72%	
Survival status (n/%)			0.046*
Alive	44/44.90%	15/28.30%	
Dead	54/55.10%	38/71.70%	
Survival time/years	1.731 ± 1.728	1.300 ± 1.351	0.016*
FAB subtype (n/%)			0.001***
M0	1/1.02%	15/28.30%	
M1	22/22.45%	13/24.53%	
M2	29/29.59%	9/16.98%	
M3	15/15.30%	0/0%	
M4	22/22.45%	7/13.21%	
M5	7/7.14%	8/15.09%	
M6	2/2.04%	0/%	
M7	0/0%	1/1.89%	
CLAGB (n/%)			0.002***
Poor	26/26.53%	29/54.72%	
Intermediate/Normal	50/51.02%	19/35.85%	
Favorable	22/22.45%	5/9.43%	

CLAGB, Cancer and Leukemia Group B.

*p < 0.05; ***p < 0.001.

### Immunological Characteristics of Pyroptosis-Related Clusters in Acute Myeloid Leukemia

**Figure 4:**
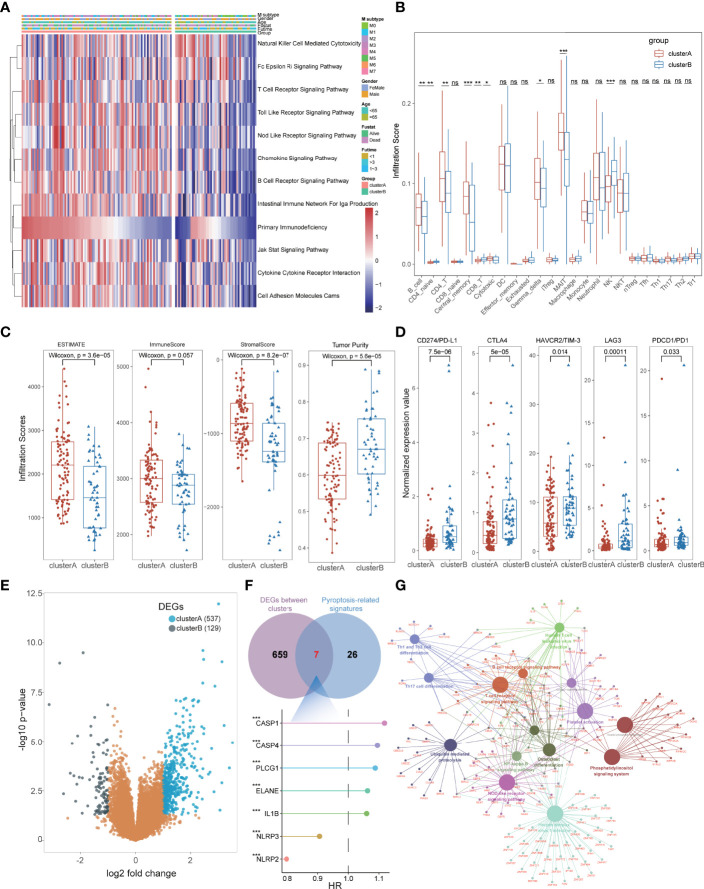
The immunological characteristics of subtypes in AML patients. **(A)** GSVA indicated that immune-related pathways were significantly activated in cluster A groups. **(B)** Massive immune cells also infiltrated the bone marrow tissues of cluster A with higher levels than those in cluster **(B, C)** TME analysis detected higher stromal and immune scores with lower tumor purity in cluster A patients. **(D)** Higher expression of immune checkpoints was found in cluster B, including PD1, PD-L1, CTLA4, HAVCR2, and LAG3. **(E)** A total of 666 DEGs were identified including 537 DEGs for cluster A and 129 DEGs for cluster **(B, F)** Seven pyroptosis-related DEGs were identified with significant prognostic value for AML patients. **(G)** KEGG enrichment analysis showed that cluster A-associated DEGs were enriched in the above immune-activated associated pathways. *P<0.05, **P<0.01, ***P<0.001; ns, not significant.

To better interpret the potential mechanism of prognostic differences in distinct pyroptosis-related subtypes in AML patients, we further explored their immunological characteristics respectively, including GSVA, ICI, TME, and immune checkpoint analysis. GSVA revealed that most immune-related pathways were significantly activated in cluster A groups such as Natural Killer Cell-Mediated Cytotoxicity, T-Cell Receptor Signaling Pathway, Chemokine Signaling Pathway, B-Cell Receptor Signaling Pathway, JAK-STAT Signaling Pathway, and Cytokine–Cytokine Receptor Interaction (**Figure 4A**; **Supplementary Table S6**). For the results of ICI analysis, massive immune cells also infiltrated the bone marrow tissues of cluster A with higher levels than that in cluster B, including B cells, CD4+ T cells, Gamma-delta T cells, MAIT cells (**Figure 4B**; **Supplementary Table S7**). TME analysis also detected higher stromal and immune scores with lower tumor purity in cluster A patients, consistent with its immune-activated status and better prognosis (**Figure 4C**; **Supplementary Table S8**). Notably, higher expression of these immune checkpoints, including PD1, PD-L1, CTLA4, HAVCR2, and LAG3, was found in cluster B, suggesting that these cases might be more sensitive to immunotherapy (**Figure 4D**; **Supplementary Table S9**). All these results conformably expounded the immune-activated condition of cluster A subtypes and macroscopically interpreted its better prognosis of AML. Furthermore, we performed differential expression analysis between the two subtypes, and a total of 666 DEGs were identified including 537 DEGs for cluster A and 129 DEGs for cluster B (**Figure 4E**, **Supplementary Table S10**). Combined with the above 33 PRGs, a total of 7 pyroptosis-related DEGs were further identified with significant prognostic value for AML patients, of which five genes (CASP1, CASP4, PLCG1, ELANE, and IL1B) were pathogenic genes and two (NLRP2 and NLRP3) had protective roles (**Figure 4F**). The Kyoto Encyclopedia of Genes and Genomes (KEGG) enrichment analysis also validated that cluster A-associated DEGs were enriched in the above immune-activated associated pathways (**Figure 4G**; **Supplementary Table S11**), and the cluster B-related DEGs were enriched in transcription-related biological processes such as DNA replication, Cell cycle, and Protein processing in endoplasmic reticulum (**Supplementary Figure S1D**).

### Construction and Prognostic Characteristics of Pyroptosis-Related Gene Scores

**Figure 5:**
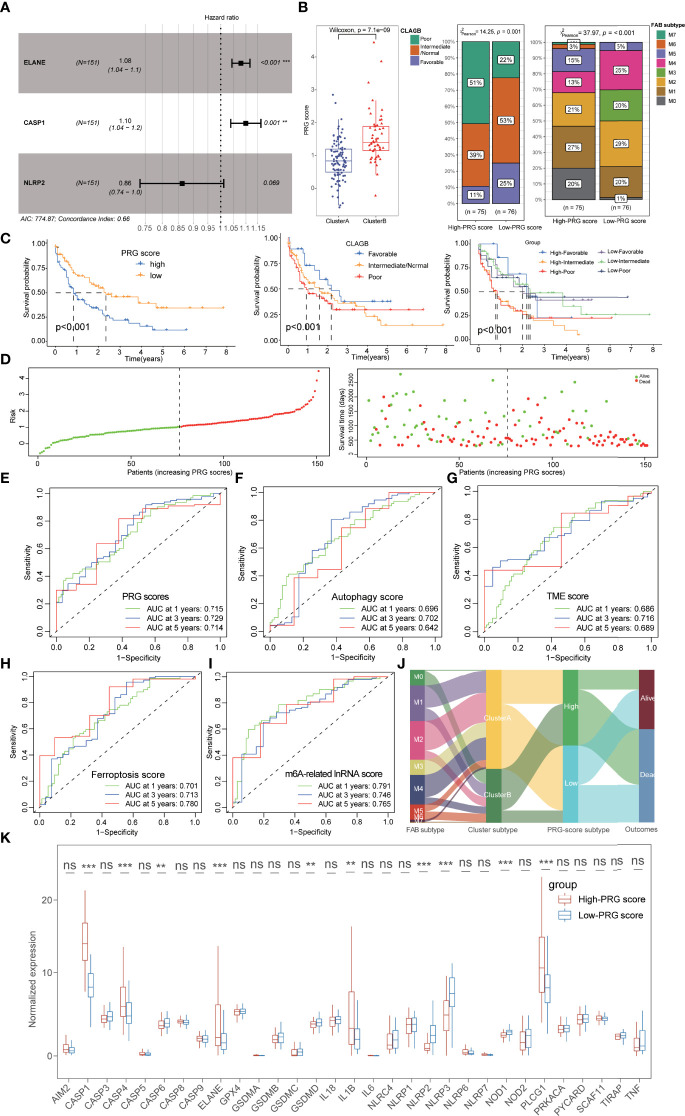
Construction and prognostic characteristics of PRG scores. **(A)** Three PRGs (ELANE, CASP1, and NLRP2) were identified by multivariate Cox regression analysis. **(B)** Cluster A possessed lower PRG scores than those of cluster B patients, and the low-PRG score patients exhibited a longer survival time with better FAB subtypes and CLAGB outcomes. **(C)** The stratified survival analysis based on PRG scores and CLAGB outcomes revealed that patients with low PRG scores had a better prognosis regardless of different outcomes. **(D)** The risk of death in AML patients also increased along with the increase of PRG scores. **(E)** ROC analysis revealed that the 1-,3-, and 5-year AUC values of the PRG score were 0.715, 0.729, and 0.714. **(F–I)** ROC analysis compared the 1-, 3-, and 5-year AUC values of multiple previous signatures for AML, with 0.696/0.702/0.642 in autophagy score **(F)**, 0.686/0.716/0.689 in TME score **(G)**, 0.701/0.713/0.780 in ferroptosis score **(H)**, and 0.791/0.746/0.765 in m6A-related lncRNA score **(I)**. **(J)** The alluvial diagram visualized the status changes in patients’ different subtypes. **(K)** The boxplots showing the significant expressional differences of PRGs between high- and low-PRG score groups. *P<0.05, **P<0.01, ***P<0.001; ns, not significant.

Based on the expression of 7 OS-associated genes, we further conducted the multivariate Cox regression analysis to obtain the three ultimate PRGs (ELANE, CASP1, and NLRP2) with the minimal Akaike information criterion (AIC) value in stepwise model, including two pathogenic genes and one favorable gene (**Figure 5A**; **Supplementary Table S12**). Using the regressive coefficients of OS-associated PRGs, the PRG score was successfully established according to the following formula:


PRGscore=Exp(ELAINE)∗0.077+Exp(CASP1)∗0.095+Exp(NLRP2)∗(−0.148)

Interestingly, we also observed a significant correlation between PRG scores and pyroptosis-related subtypes and found that cluster A possessed lower PRG scores than that of cluster B patients (**Figure 5B**). Subsequently, 151 AML patients were separated into the high- and low-PRG score cohorts with the median value (1.035) as the cutoff value, and the low-PRG score patients exhibited a longer survival time with better FAB subtypes and CLAGB outcomes (**Figures 5B, C** **)**. Moreover, the stratified survival analysis based on PRG scores and CLAGB outcomes revealed that patients with low PRG scores had a better prognosis regardless of different outcomes, suggesting that the PRG score was an independent prognostic element for AML (**Figure 5C**). The risk of death in AML patients also increased along with the increase of PRG scores (**Figure 5D**), and ROC analysis revealed the 1-, 3-, and 5-year AUC values of the PRG score were 0.715, 0.729, and 0.714, respectively (**Figure 5E**). Moreover, the PRG score also exhibited excellent prognostic value compared with a previously published index for AML, including 1-, 3-, and 5-year AUC values 0.696/0.702/0.642 of autophagy score, 0.686/0.716/0.689 of TME score, 0.701/0.713/0.780 of ferroptosis score, and 0.791/0.746/0.765 of m6A-related lncRNA score (**Figures 5F–I**). The alluvial diagram visualized the status changes in patients’ different subtypes, and it revealed the consistency of cluster A and low-PRG score subtypes with better survival (**Figure 5J**). Significant expressional differences of PRGs were also exhibited between high- and low-PRG score groups, consistent with the difference between pyroptosis-related clusters (**Figure 5K**).

### Immunological Characteristics and External Validation of Pyroptosis-Related Gene Scores

**Figure 6:**
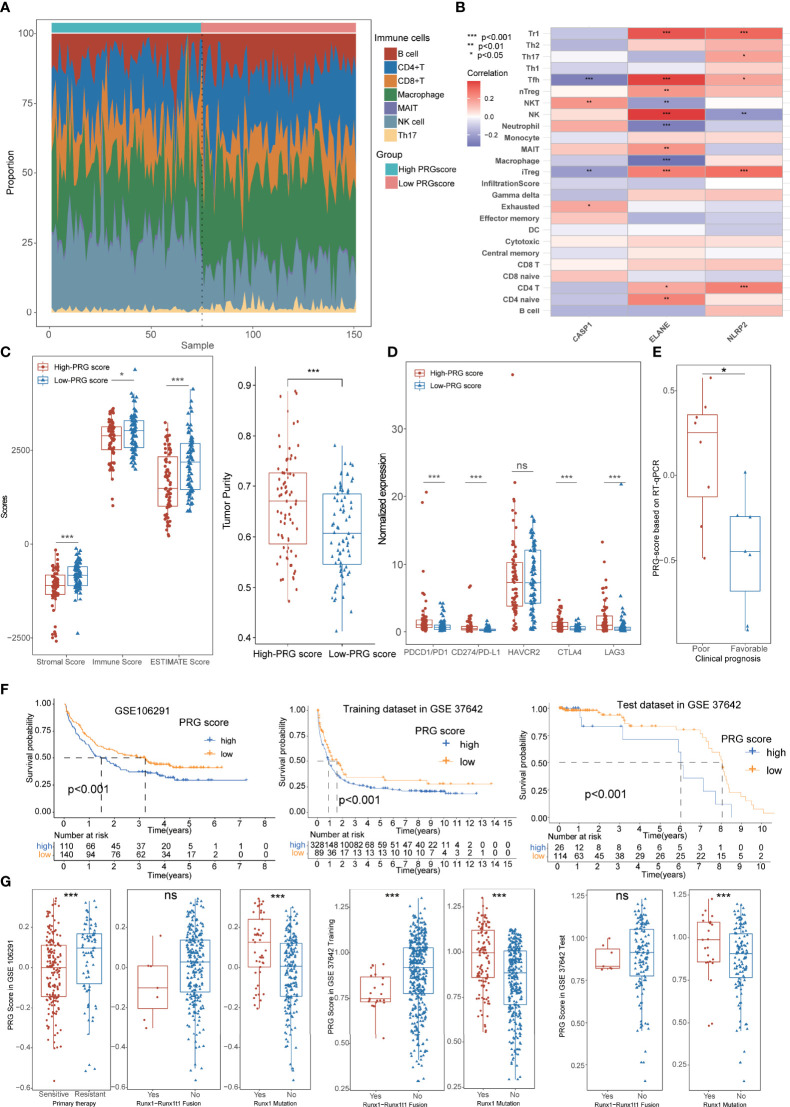
Immunological characteristics and external validation of PRG scores. **(A)** The scale diagram showing the different infiltrate levels of immune cells between high- and low-PRG score groups. **(B)** Correlation analysis of immune cells and PRGs. **(C)** TME analysis showed that the low-PRG score patients showed higher immune and stromal scores with lower tumor purity than those of high-PRG score patients. **(D)** Higher expression levels of immune checkpoints were identified in high- and low-PRG score groups. **(E)** AML patients with favorable prognosis exhibited lower PRG scores than those of poor-prognosis patients using RT-qPCR data. **(F)** Survival analysis validated a better prognosis in the low-PRG score group in external datasets. **(G)** The boxplots showed that patients with Runx1 mutation exhibited higher PRG scores, while patients with Runx1-Runx1t1 fusion exhibited a tendency to have lower PRG scores. *P<0.05, **P<0.01, ***P<0.001; ns, not significant.

We also performed an immunological analysis to explore the potential correlation of immune characteristics and PRG scores, including ICI, TME, and immune checkpoint analysis. Massive immune cells infiltrated in the low-PRG score cohorts, and those immune cells exhibited significant association with PRGs, especially ELANE (**Figures 6A, B**; **Supplementary Figure S3A**; **Supplementary Table S13**). TME analysis also demonstrated that the low-PRG score patients showed higher immune and stromal scores with lower tumor purity than that of high-PRG score patients (**Figure 6C**). Notably, the higher expression levels of immune checkpoints were also identified in high-PRG score groups, implying its potential sensitivity to immunotherapy (**Figure 6D**). All these pieces of evidence conformably indicated that the low-PRG score groups, consistent with cluster A subtypes, possessed an immune-activated status and better prognosis for AML.

To further estimate the prognostic value of the PRG scores in external datasets, we recalculated the score based on the expression of 3 PRGs from our RT-qPCR data and five GEO datasets and performed a corresponding clinical analysis. Based on the comprehensive clinical evaluations, AML patients with favorable prognosis exhibited lower PRG scores than those of poor-prognosis patients (**Figure 6E**; **Supplementary Tables S14, 15**), and there was no significant association between PRG scores and common clinical features including age, gender, and onset time (**Supplementary Figure S3C**). All of the results of the survival analysis validated a better prognosis in the low-PRG score group (**Figure 6F**, **Supplementary Figure S3D**). Moreover, the prognostic value of PRG scores was further validated in external test datasets, including 1-, 3-, and 5-year AUC values 0.635/0.645/0.736 in GSE106291, 0.581/0.564/0.613 in GSE37642 training sets, 0.781/0.713/0.594 in GSE37642 test sets, 0.645/0.709 in GSE12417 training sets, and 0.739/0.747 in GSE12417 test sets (**Supplementary Figure S3E**). Interestingly, mutational information of Runx1 was also recorded in the external datasets, and the boxplots showed that patients with Runx1 mutation exhibited higher PRG scores, which reflected worse prognosis in clinical practice. Contrarily, patients with Runx1-Runx1t1 fusion, reflecting better survival in practice, exhibited a tendency of lower PRG scores (**Figure 6G**).

### Evaluation of Therapeutic Susceptibility and Development of a Prognostic Model for Acute Myeloid Leukemia

**Figure 7:**
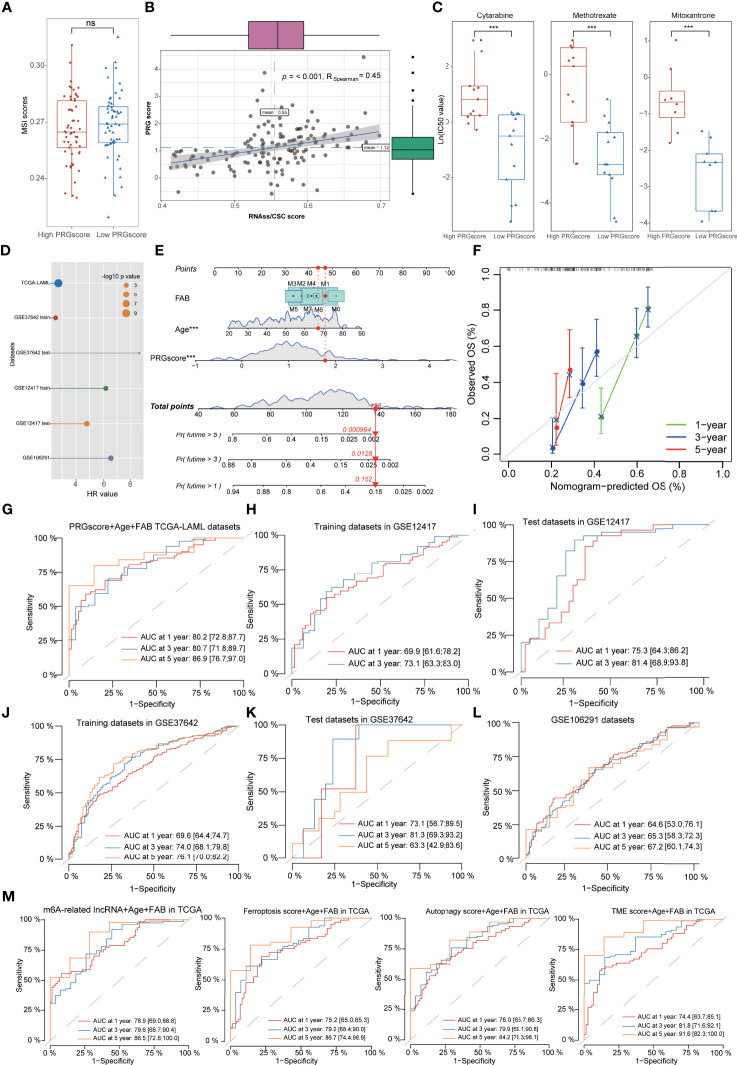
Evaluation of therapeutic susceptibility and development of a prognostic model for AML. **(A)** There was no difference of MSI scores between high- and low-PRG score patients. **(B)** The correlation analyses revealed that PRG scores were significantly positively associated with mRNAsi. **(C)** The anti-AML chemotherapeutic drugs (cytarabine, methotrexate, and mitoxantrone) exhibited lower IC50 values in low-PRG score groups. **(D)** Univariate Cox regression analysis demonstrated that PRG scores could serve as an independent risk factor for the prognosis of AML with high HR values in multiple datasets. **(E)** A combined nomogram for predicting the probability of 1-/3-/5-year survival for AML patients. **(F)** The calibration curve of the established nomogram with 1-/3-/5-year survival, respectively. **(G–L)** Time-dependent receiver operating characteristic (ROC) curves of 1-/3-/5-year survival for AML patients in TCGA, GSE12417 training sets, GSE12417 test sets, GSE37642 training sets, GSE37642 test sets, and GSE106291 datasets. **(M)** Time-dependent ROC analysis of 1-/3-/5-year survival for AML patients using the gene models with existing signatures, including m6A-related lncRNA models, ferroptosis-related models, autophagy-related models, and TME score models. *P<0.05, **P<0.01, ***P<0.001; ns, not significant.

Increasing evidence has proven that patients with low mRNAsi and high MSI values are generally sensitive to available therapies (39, 40). In this study, we did not detect the difference of MSI scores between high- and low-PRG score patients, but the correlation analyses revealed that PRG scores were significantly positively associated with mRNAsi (R = 0.45, p < 0.001; **Figures 7A, B**; **Supplementary Table S16**). For drug susceptibility, the therapeutic responses to primary “7+3” chemotherapy were recorded in GSE106291 and revealed that patients sensitive to chemotherapy possessed lower PRG scores compared with resistant AML patients (**Figure 6F**). Moreover, anti-AML chemotherapeutic drugs (cytarabine, methotrexate, and mitoxantrone) also exhibited lower IC50 values in low-PRG score groups, indicating that these subtypes might obtain a better curative efficacy from the chemotherapy (**Figure 7C**).

Univariate Cox regression analysis demonstrated that PRG scores could be served as an independent risk factor for the prognosis of AML with high hazard ratio (HR) values in multiple datasets (**Figure 7D**, **Supplementary Table S17**). Based on the PRG scores and important clinical characteristics, a nomogram was constructed using multivariate Cox model to accurately predict the probability of 1-/3-/5-year survival for AML patients. FAB subtypes, age, and PRG scores were included in the nomogram (**Figure 7E**), and calibration curves exhibited that the nomogram had a good prediction capacity for AML patients (**Figure 7F**). The ROC analysis revealed that the 1-, 3-, and 5-year AUC values of the nomogram were 0.802, 0.807, and 0.869, respectively (**Figure 7G**). The external datasets further validated the predictive potential of the nomogram for the prognosis of AML, including 0.699/0.731 in GSE12417 training sets, 0.753/0.814 in GSE12417 test sets, 0.696/0.740/0.761 in GSE37642 training sets, 0.731/0.813/0.633 in GSE37642 test sets, and 0.646/0.653/0.672 in GSE106291 datasets (**Figures 7H–L**, **Supplementary Table S18**). Notably, compared with the gene model with existing signatures, our nomogram system also displayed relatively promising predictive capability such as the 1-/3-/5-year AUC values 0.789/0.796/0.865 in m6A-related lncRNA models, 0.752/0.792/0.867 in ferroptosis-related models, 0.760/0.799/0.842 in autophagy-related models, and 0.744/0.818/0.916 in TME score models (**Figure 7M**).

## Discussion

As a common malignant tumor with a rapid progress and high mortality (the 5-year survival is <30%), AML has been known for its poor prognosis with drug resistance and recurrence due to abnormal molecular and genomic changes (41). More and more publications have demonstrated that current conventional intensive chemotherapy failed to fully satisfy the extremely complicated heterogeneity in AML patients, indicating the unmet need for new prognostic biomarkers to improve the precision of AML stratification and treatment (42, 43). As the only potentially curable option for AML patients, allogeneic hematopoietic stem cell transplantation (allo-HSCT) achieves most durable remissions for high-risk patients, but it still faces several severe challenges due to inner heterogenicity, especially graft-versus-host disease (GVHD) and disease relapse. Approximately 40% of AML patients with allo-HSCT would relapse and exhibit a terrible prognosis with <20% of 2-year survival (44). Recently, the chimeric antigen receptor T (CAR-T) cell therapy has brought a new breakthrough to the treatment of hematological cancers, especially for therapy-resistant and refractory AML patients, with advantages of specific major histocompatibility complex (MHC)-independent antigen recognition, higher proliferation, and manageable cytotoxic capacity (45). The CAR-T cells are regularly engineered to target multiple myeloid-lineage antigens such as CD33, CD123, and CD7, but the off-target antigen toxicity to other organs is still the prominent challenge and remains to be further predicted, especially in patients with complex disease stages of cellular differentiation (46–48). In addition, more and more research focuses on the exploration of various novel target antigens utilizable for engineering CAR-T cells against various subtypes of AML. For example, Jetani etal. (49) successfully engineered CAR-T cells targeting FMS-like tyrosine kinase 3 (FLT3) to treat the high-risk FLT3-ITD+ AML patients synergistically with the FLT3 inhibitor crenolanib. Therefore, identification of a novel molecular subtype and reliable objective index for predicting curative effects and prognosis in AML patients is urgently needed.

Different from apoptosis, pyroptosis has been considered as a positive PCD process and usually occurs in abnormal infected cells or tumor cells, thus inducing the release of pro-inflammatory cytokines and the activation of the inflammatory response (50). With the activation of stress, such as infection, tumors, and other factors, pyroptosis can also be converted from apoptosis and participate in the complex process of multiple tumors. Publications have demonstrated that pyroptosis could play its antitumor effectiveness by inhibiting tumor growth in hepatocellular and gastric carcinoma while with an inhibitory or promotion two-way efficacy in breast cancers (51–53). Moreover, Johnson etal. (9) also demonstrated that a DPP8/DPP9 inhibitor could induce pyroptosis to ameliorate AML *via* pharmacological intervention experiments *in vitro*. Generally, the activation of NLRP (NOD-, LRR-, and pyrin domain-containing) inflammasome was classically integrant for the activation of pyroptosis through recruiting CASP1, further inducing the cleavage of GSDMD (16). Interestingly, our results also detected a higher expression of PRGs in AML patients including NLRP/CASP/GSDM families, and these PRGs were also significantly enriched in NOD-like signaling pathway and immunoactivated-related pathways, implying that pyroptosis might participate in the progression of AML and was associated with the prognosis of AML.

Multiple genetic alterations and molecular genetic analyses have provided useful information for predicting the risk stratification and prognoses of AML patients, especially somatic mutation and copy number variations (CNVs) (54, 55). In this study, we also investigated the genetic characteristics of PRGs in AML patients, but it revealed that somatic mutation of PRGs was detected in only 3% of cases and the maximum CNV frequency was only 2.5% for PRGs, suggesting that pyroptosis might be independent of genetic mutation in AML patients. The classification of AML patients based on various pathognomonic gene expression profiles has been considered a promising method and applied to various studies including the immune microenvironment (11), autophagy-related signatures (12), and N6‐methyladenosine (13). Our study first proposed a pyroptosis-related molecular subtype based on clustering PRGs with distinct clinical prognostic and immunological characteristics including TME, ICI, and immune checkpoints. Notably, cluster A presented a longer median survival time than cluster B, and the prognostic subtypes were consistent with clinical risk stratifications including FAB subtypes and CLAGB outcomes, indicating that these PRGs were also significantly associated with survival risks in AML patients. It was worth noting that the clustering subtypes reached some consensuses: 1) the pyroptosis-related signatures exhibited complicated expression levels between clusters such as lower levels of CASP1/4 and higher levels of ELANE and NLRP3 in cluster A; 2) Cluster A was a specific phenotype with a better prognosis and slighter clinical FAB phenotypes; 3) Cluster A was identified as an immune-activated subtype with higher TME scores and infiltration degree of adaptive immune response-related immune cells, while cluster B exhibited potential sensitivity to PD-L1 treatment in AML.

Tumor cells manipulate and reshape the TME into a pro-leukemia phenotype to promote AML progression through a complex interaction network, including apoptosis resistance, proliferation acceleration, and malignant metastasis (56). Meanwhile, immune cells and stromal cells in the TME in turn played essential protective and regulatory effects on AML, especially *via* an immune cell-induced inflammatory response (57). These studies indicated the existence of close association between TME and the prognosis of AML. In this study, we also applied the ESTIMATE algorithm to estimate the immune scores, stromal scores, and tumor purity of all AML samples, and results revealed higher immune and stromal scores and lower tumor purity in cluster A than those of cluster B cohorts, suggesting the consistency between TME scores and prognostic outcomes of AML. Moreover, we also detected the negative correlation of PRG scores and TME scores, and the low-PRG score subgroups exhibited a longer median survival time than that in high-PRG score cohorts, indicating the inner connection between pyroptosis and TME characteristics in AML.

CAR-T cell therapy has brought a new breakthrough for the treatment of AML and proves that the application of normal immune cells to change and reshape the abnormal immune microenvironment is valid and feasible. To systematically evaluate the immune microenvironment of AML, we further used the CIBERSORT algorithm to identify the infiltration scores of various immune cells and explore their prognostic capacity and relationship with PRGs. Interestingly, ICI analysis revealed that substantial immune cells were significantly activated in cluster A groups including CD8+ T cells, CD4+ central memory T cells, and B lymphocytes, demonstrating the immune activation status in cluster A subtypes. Notably, several special T-lymphocyte subtypes also significantly infiltrated cluster A cases, including CD8+ cytotoxic T cells and γδT cells, coinciding with the findings of previous studies. For example, Halim etal. (58) also found increased γδT cells might be the most prognostically favorable immune-cell infiltration with special MHC antigen-presenting in AML. In addition, Garcia-Guerrero etal. (59) successfully extracted tumor-specific cytotoxic T lymphocytes against AML blasts from AML patients based on fluorescence-activated cell sorting (FACS) technique. These studies were consistent with the results that proportions of ICI of cytotoxic T cells and γδT cells exhibited a better outcome of cluster A and low-PRG score AML patients in this study.

A novel pyroptosis-related scoring tool (PRG score) was successfully constructed to determine the prognostic risk of AML based on the multivariate Cox regression (stepwise model) of differentially expressed PRGs from two clusters. Interestingly, higher infiltration of immune cells, TME scores, and better survival status were detected in low-PRG score groups, consistent with the characteristics of cluster A cohorts. Moreover, the association between PRG score and clinical prognosis status was further validated *via* RT-qPCR data in this study. Notably, the PRG score was calculated based on the expression of CASP1, ELANE, and NLRP2, all associated with the process of AML according to previous research. As a member of cysteine acid proteases family, high expression of CASP1 has been demonstrated to be positively associated with poor prognosis in AML patients and CASP1 inhibition could obviously inhibit the proliferation of AML cells, implying that CASP1 might serve as a potential biomarker to predict the prognosis and therapeutic target for AML patients (60). In another study of pyroptosis, NLRP2 was also used to identify the PRG score for predicting the prognosis of lung adenocarcinoma (61), and Li etal. (62) further verified its inhibitory effect on cell proliferation and migration *via* inducing epithelial-to-mesenchymal transition (EMT) in lung adenocarcinoma cell lines, suggesting that NLRP2 might serve as a tumor suppressor. Another large-scale DNA methylation transcriptome analysis also identified the neutrophil-expressed elastase (ELANE) as a vital biomarker associated with the invasion and metastasis of clear cell renal cell carcinoma (ccRCC) (63). The prognostic capability of PRG scores was further validated in external test datasets, and the parameter still exhibited an excellent prognostic value compared with a previously published index for AML. These results imply the PRG’s potential association with the prognosis of AML, and the concrete mechanism of these vital pyroptosis-related signatures in AML is worth to be further investigated by functional experiments *in vivo* and *in vitro*.

MSI refers to a hypermutable feature caused by the loss of DNA mismatch repair (MMR) and has been acknowledged as a prognostic biomarker for the treatment of multiple tumors, indicating that MSI could serve as an essential index associated with DNA mutation (64). Cancer stem cells (CSCs) represent the potential origin of cancers, and a higher leukemia stem cell (LSC) proportion had been reported to display a worse prognosis with shorter relapse-free survival (RFS) in AML patients (65). As the most representative parameter of CSC, the mRNAsi has been widely applied to evaluate CSC characteristics in various tumors including AML (40). In this study, we also investigated the correlation of MSI scores, mRNAsi, and PRG scores, and our results exhibited that there was no significant relationship between MSI and PRG scores, consistent with previous findings of somatic mutation and CNV analysis. However, the mRNAsi possessed a significantly positive correlation with PRG scores and supported their common prediction ability for the prognosis of AML. For drug sensitivity, our findings showed higher IC50 values of chemotherapy drugs in AML patients with high PRG scores, implying that those patients might be resistant to common chemotherapy. Datasets from GSE106291 also validated the negative relationship between high PRG scores and drug resistance. Finally, combined with age, FAB subtypes, and PRG scores, we further established a useful nomogram scoring system to accurately predict the 1-/3-/5-year survival of AML, and the model was validated in multiple external datasets with a high AUC value. Notably, our nomogram system still displayed a relatively promising predictive capability for AML compared with the gene models with existing signatures, including m6A-related lncRNA models, ferroptosis-related models, autophagy-related models, and TME score models.

However, there are still some inescapable limitations in our study. For one thing, the initial and validation analysis based on the RNA-seq profiles was relatively insufficient because it was just obtained from public databases. Although we have performed the RT-qPCR experiments to validate the PRG scores, we still failed to prove the survival value due to lack of survival data in our data. Due to the limitation of corresponding data, we could not perform the survival analysis based on other types of outcomes, such as progression-free survival (PFS) and disease-free survival (DFS). These corresponding finding and conclusion remain to be further explored through more external congeneric research and validated *via* experiments *in vivo* and *in vitro*. In addition, the application of several findings in this study still needs other studies, even clinical practices, to be repetitively affirmed and improved, such as the clinical application of pyroptosis-related clusters and the concrete mechanism of PRG scores in predicting the prognosis for AML. Due to lack of clinical data of patients who received immunotherapy, we also failed to estimate the predictive role of this model for AML patients.

## Conclusion

In conclusion, our study firstly proposed a novel molecular subtype based on the clustering expression of PRGs with distinct clinical and immunological signatures in AML patients. Moreover, we identified and validated the PRG score as an effective tool to predict the OS and potential therapeutic reaction to chemotherapy for AML. Combined with age, FAB subtypes, and PRG scores, we further established a useful nomogram scoring system to accurately predict the 1-/3-/5-year survival of AML, and the model was validated in multiple external datasets with high AUC values. The various transcriptomic analyses help us screen significant pyroptosis-related signatures of AML and provide a new clinical application of PRG scores in predicting the prognosis and benefits of treatment for AML patients.

## Data Availability Statement

Publicly available datasets were analyzed in this study, and the corresponding data were displayed in the **Supplementary Material**. The data can be downloaded from here: The Genotype-Tissue Expression (GTEx) Project datasets, The Cancer Genome Atlas (TCGA) (https://portal.gdc.cancer.gov/) [GDC TCGA Acute Myeloid Leukemia (LAML)] datasets, and Gene Expression Omnibus (GEO) (https://www.ncbi.nlm.nih.gov/geo/) (Accessions: GSE106291, GSE37642, GSE12417). Other detailed data could be obtained from the corresponding author with the reasonable requirements.

## Ethics Statement

The studies involving human participants were reviewed and approved by The Ethics Committee of the First Affiliated Hospital of Wenzhou Medical University (Issuing Number: 2021063). The patients/participants provided their written informed consent to participate in this study.

## Author Contributions

JP contributed to data acquisition, analysis, figure presentation, and drafting of the article. YJ participated in the process of data acquisition and RT-qPCR experiments. CL and TJ contributed to sample collection and data analysis. KY and ZJ contributed to figure presentation, revision of the article, and the design of the study. All authors contributed to the article and approved the submitted version.

## Funding

This study was supported by the General Scientific Projects of Zhejiang Education Department under Grant Y202147905.

## Conflict of Interest

The authors declare that the research was conducted in the absence of any commercial or financial relationships that could be construed as a potential conflict of interest.

## Publisher’s Note

All claims expressed in this article are solely those of the authors and do not necessarily represent those of their affiliated organizations, or those of the publisher, the editors and the reviewers. Any product that may be evaluated in this article, or claim that may be made by its manufacturer, is not guaranteed or endorsed by the publisher.
