# Supplementary figures and images for: Characteristics of Pyroptosis-Related Subtypes and Novel Scoring Tool for the Prognosis and Chemotherapy Response in Acute Myeloid Leukemia
Source: Front Oncol. 2022 Jun 10;12:898236. doi: 10.3389/fonc.2022.898236 (PMC9229173; doi:10.3389/fonc.2022.898236)

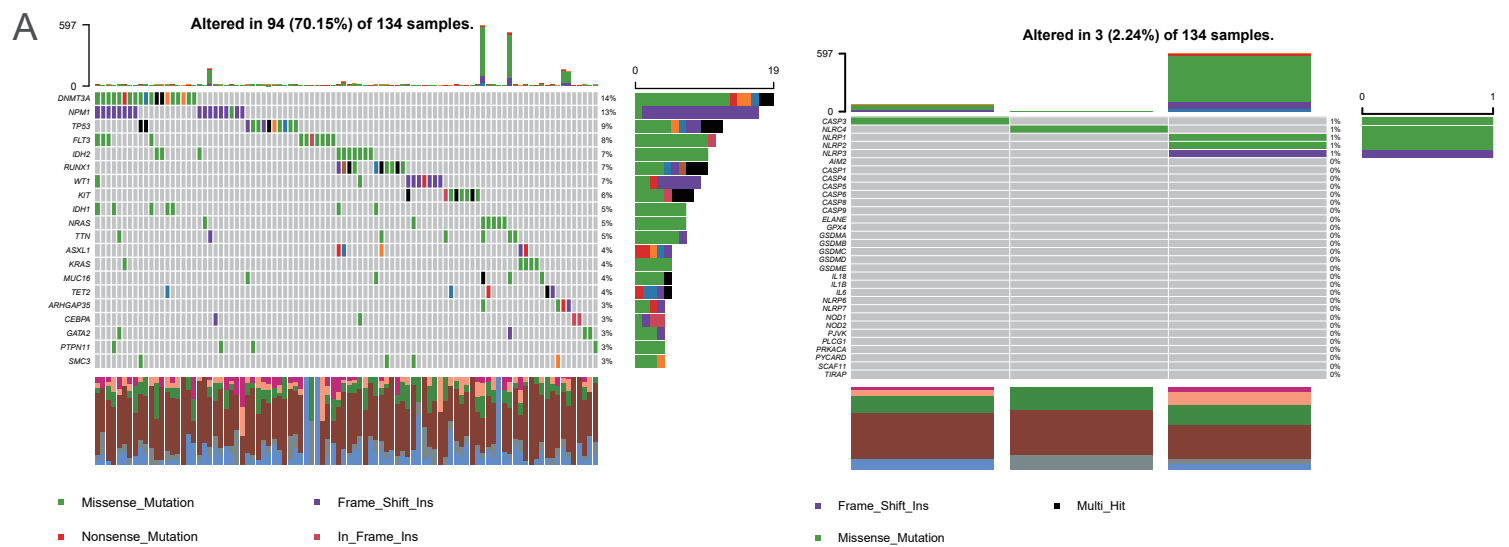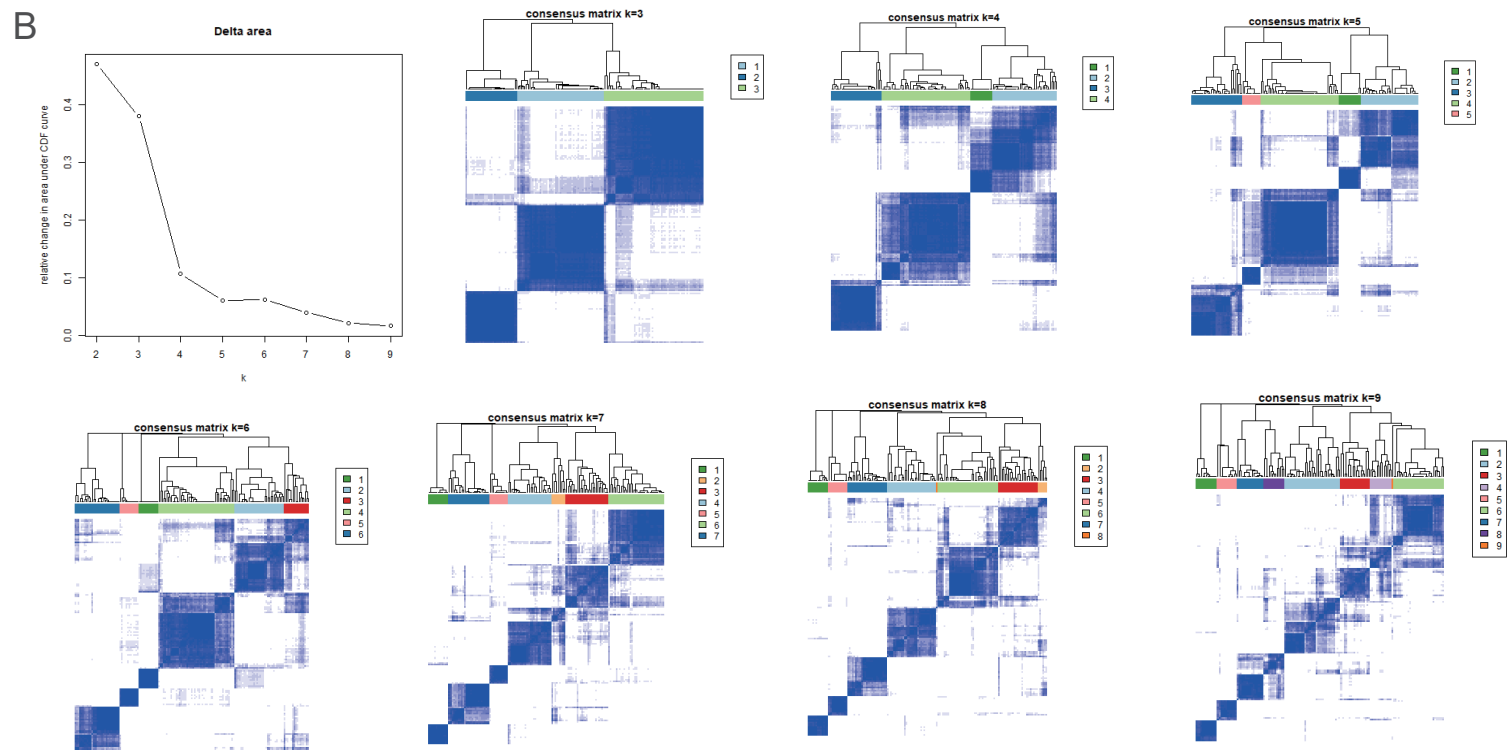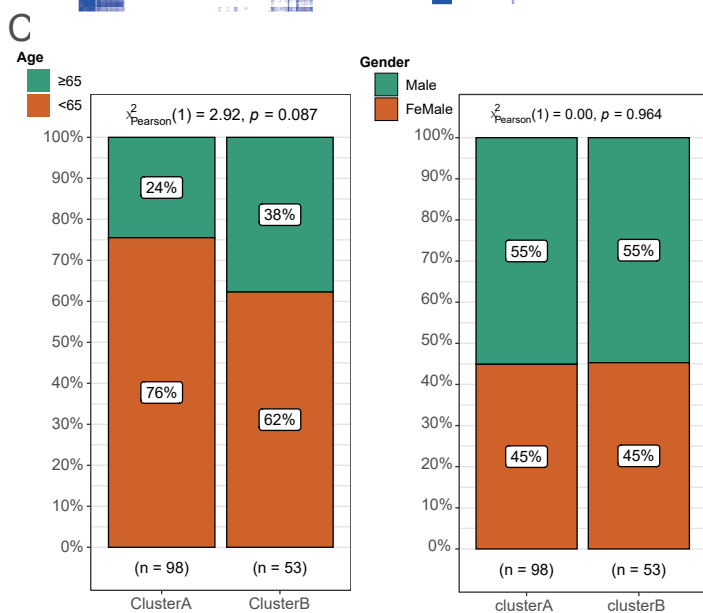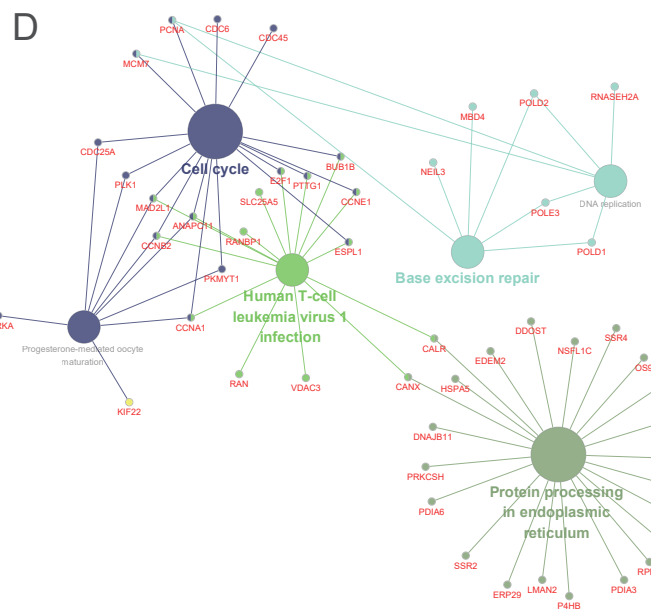

Supplement: Supplementary file 1 [file DataSheet_1.pdf]

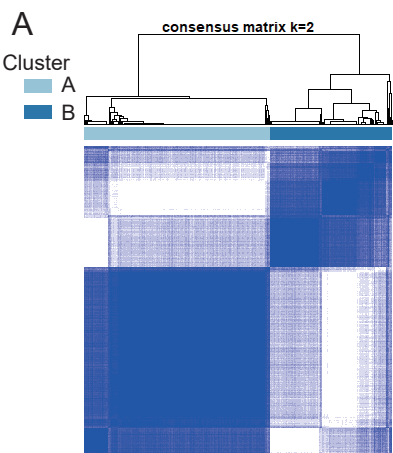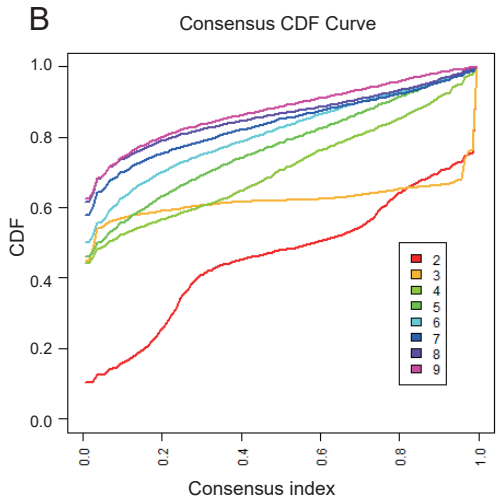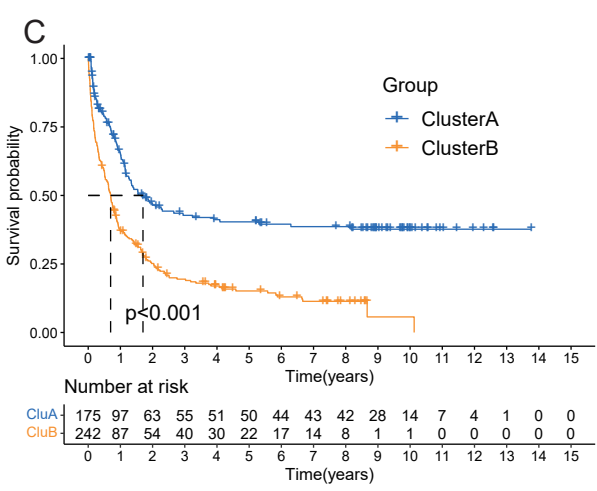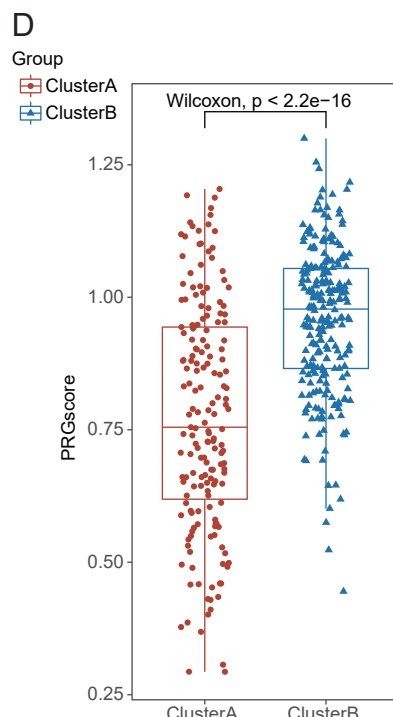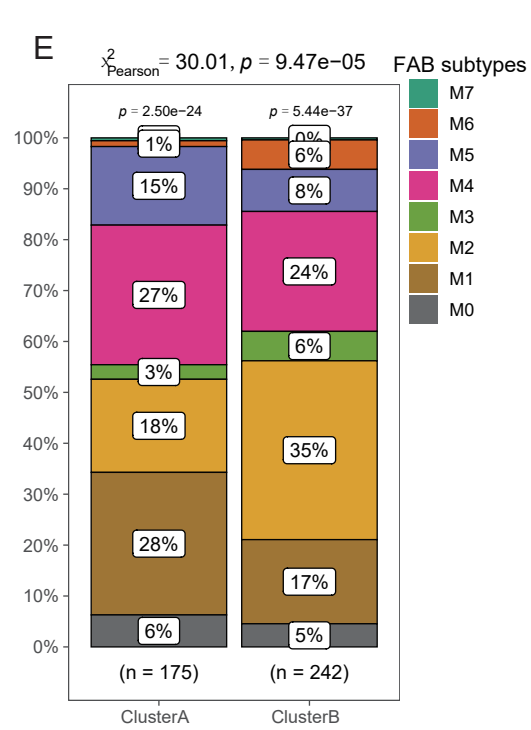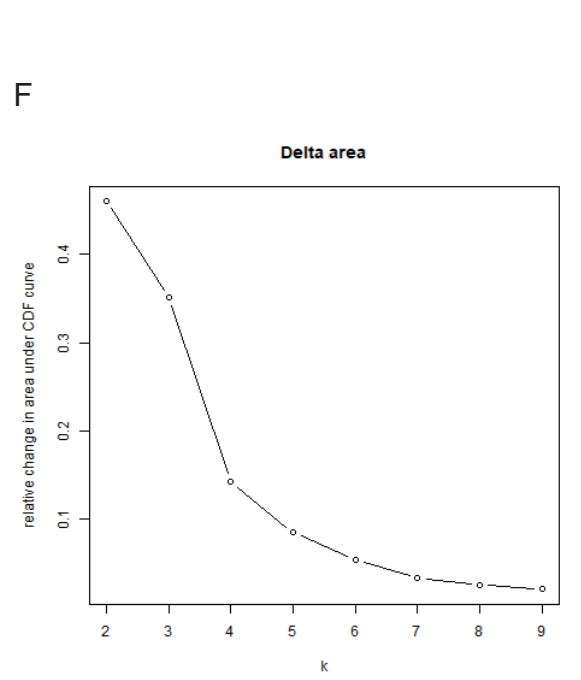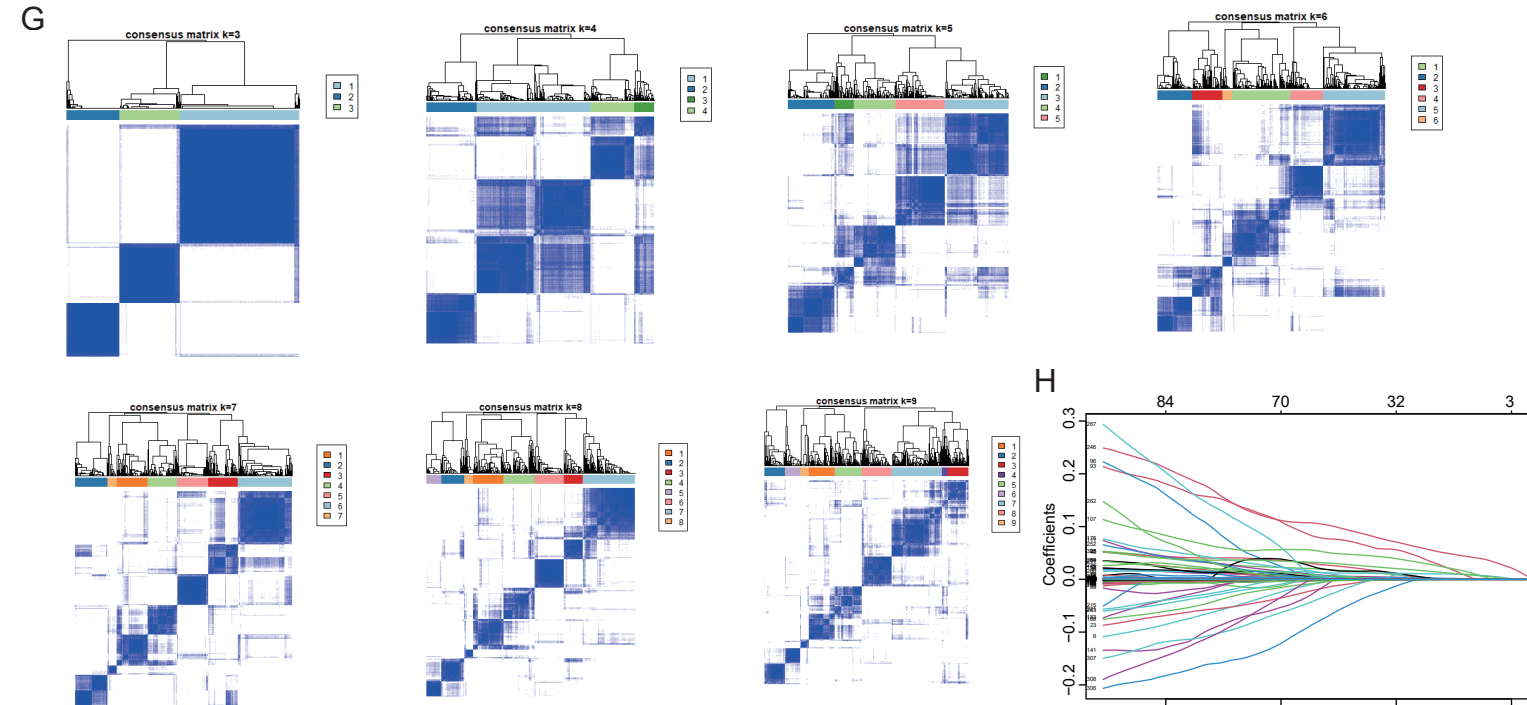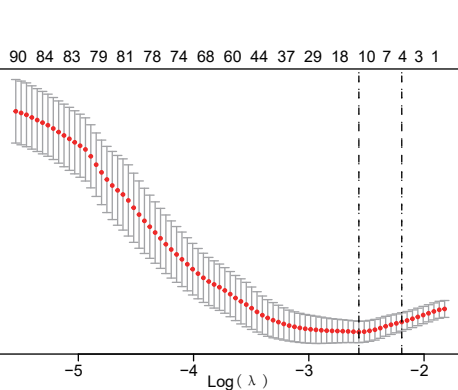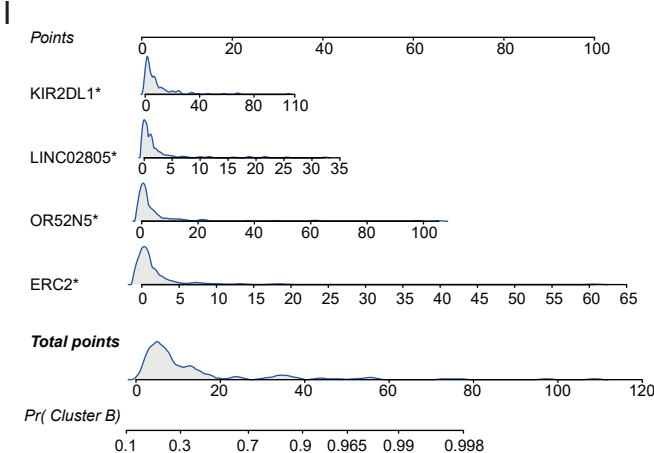

Supplement: Supplementary file 2 [file DataSheet_2.pdf]

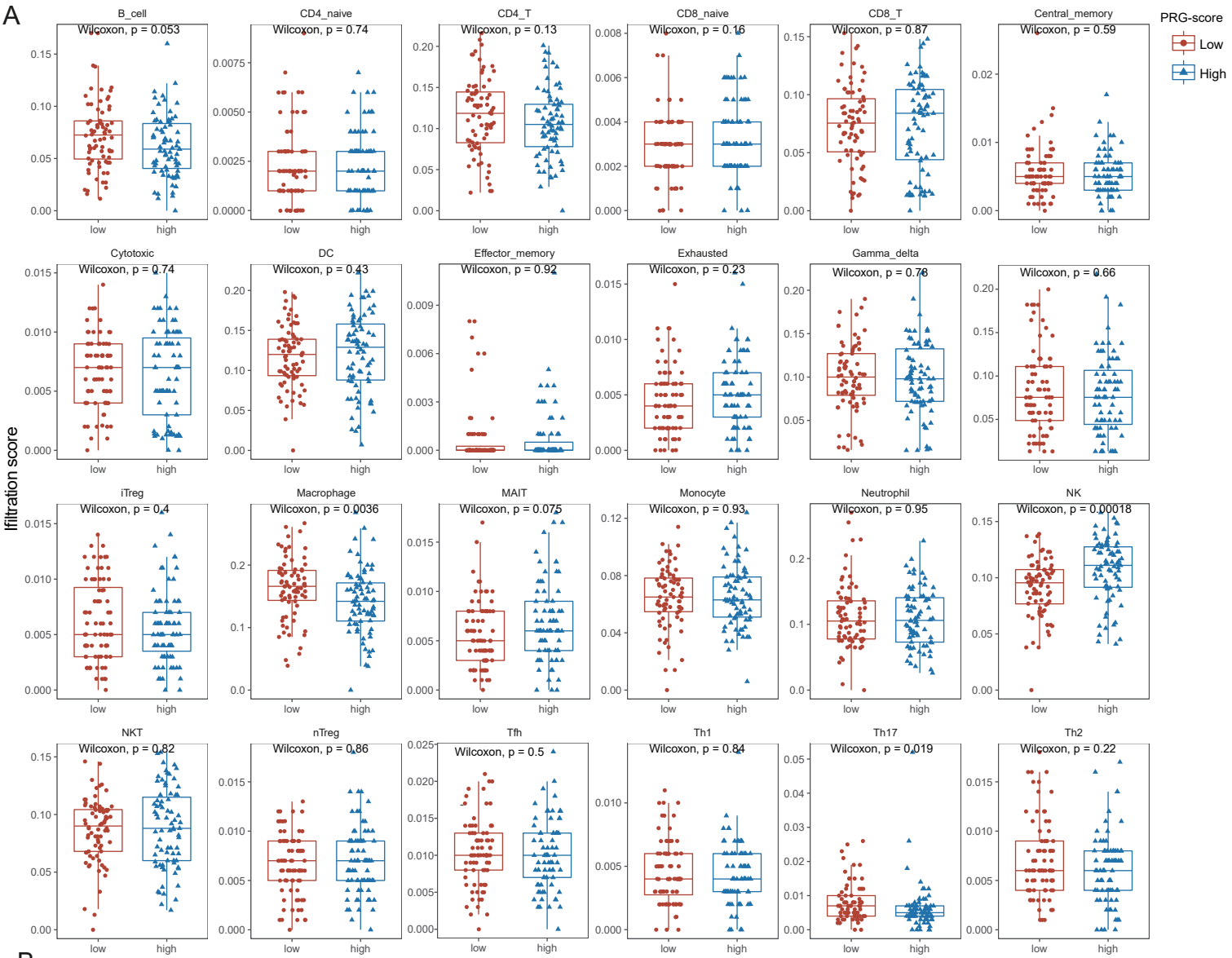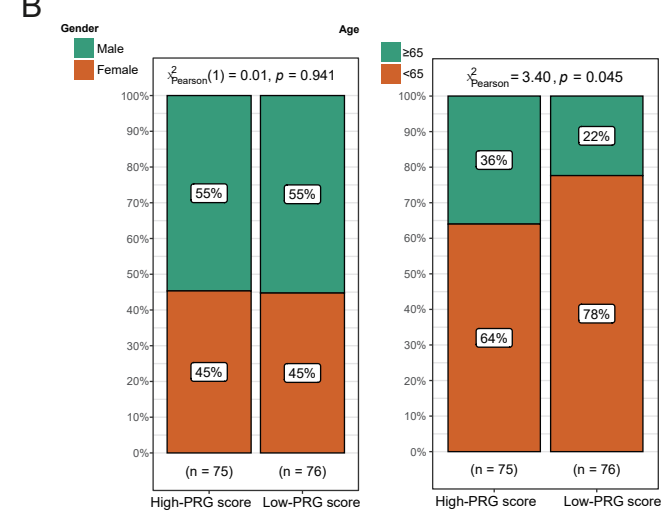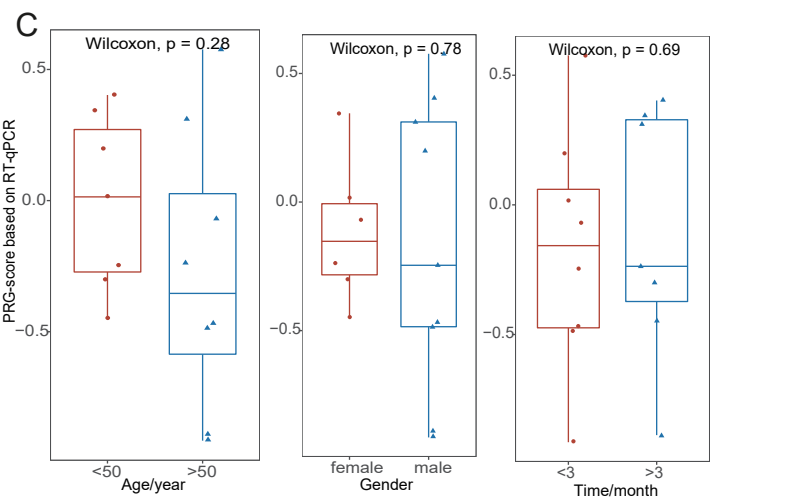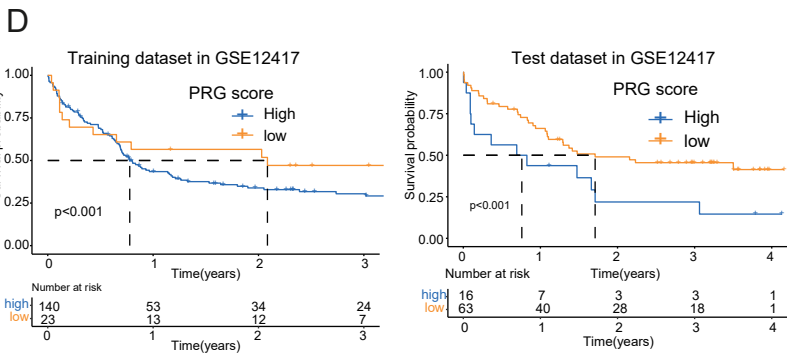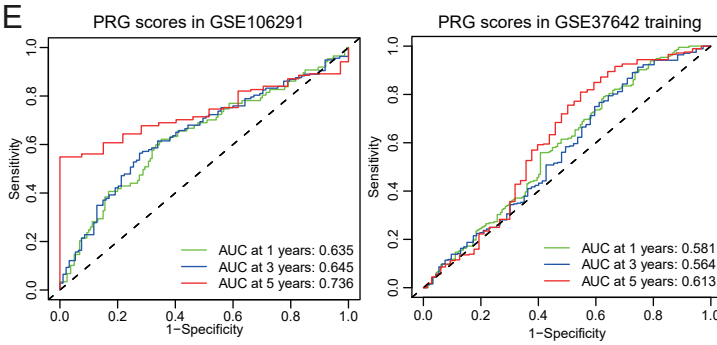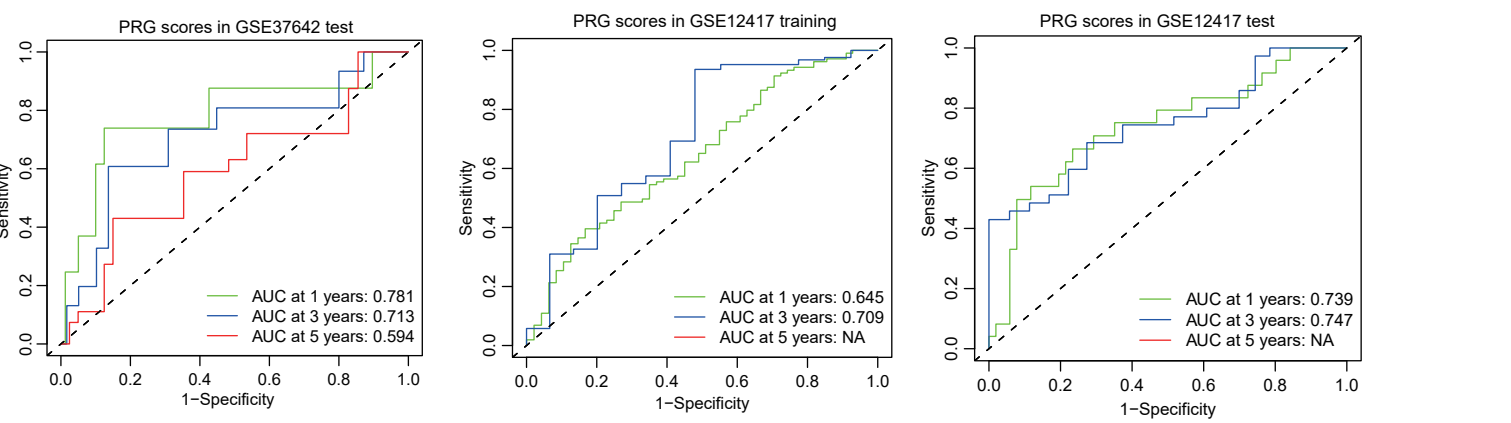

Supplement: Supplementary file 3 [file DataSheet_3.pdf]
